# Transport Properties of Self-Assembling G‑Hydrogels: Evidence for a Tunable Fickian Diffusivity

**DOI:** 10.1021/acs.jpcb.5c00564

**Published:** 2025-05-15

**Authors:** Alessia Pepe, Paolo Moretti, Paolo Mariani, Valentina Notarstefano, Francesca Ripanti

**Affiliations:** † Department of Life and Environmental Sciences, 9294 Università Politecnica delle Marche, 60131 Ancona, Italy; ‡ Department of Science and Technology for Agriculture, Food and Environment, 19031 Università di Teramo, 64100 Teramo, Italy

## Abstract

The mixing of Guanosine (Gua) and Guanosine 5′-monophosphate (GMP) in water in selected compositions yields highly hydrated, transparent, and self-healing self-assembled supramolecular G-hydrogels, attractive for biomedical applications. This work investigates how hydrogel composition affects solute transport, including diffusion, binding, loading, and release properties, using a set of fluorescent probes with varying size and polarity. Although small/wide-angle X-ray scattering techniques showed that no structural changes are induced by probe addition, even when intercalation into G-quadruplexes is expected, the internal mesh structure of the hydrogel, modulated by the Gua:GMP ratio, directly impacts probe diffusivity and loading. Tighter networks (e.g., 1:1) slow diffusion and enhance retention compared to looser configurations (e.g., 1:4). Moreover, UV–visible titrations revealed markedly different binding affinities (*K*_b_ ≈ 5.7 × 10^4^ M^–1^ for DAPI, 8.0 × 10^3^ M^–1^ for ThT, and 1.4 × 10^2^ M^–1^ for RhB), which are expected to result in lower diffusion coefficients and slower release, especially for DAPI and ThT. Indeed, diffusion coefficients, obtained via fluorescence recovery after photobleaching and time-resolved fluorescence spectroscopy, reach 90, 20, and 60 μm^2^/s for FITC-dextran, ThT, and RhB, respectively. Probe release kinetics, modeled via Weibull fitting, indicated sustained release with characteristic times (τ) between 9.6 and 23.2 h and β ≈ 1 in 1× PBS, consistent with predominantly Fickian diffusion. Remarkably, switching to 10× PBS significantly accelerated release (τ reduced by ≈ 40–50%), suggesting that ionic strength and/or pH changes critically affect not only probe-hydrogel interactions but also the internal gel architecture, altering porosity, mesh size, and network tortuosity, thus enhancing molecular mobility. Overall, the G-hydrogel system offers a structurally tunable and composition-dependent platform capable of finely regulating molecular transport and release profiles, making it highly suitable for controlled drug delivery and adaptive biomaterial applications.

## Graphical abstract

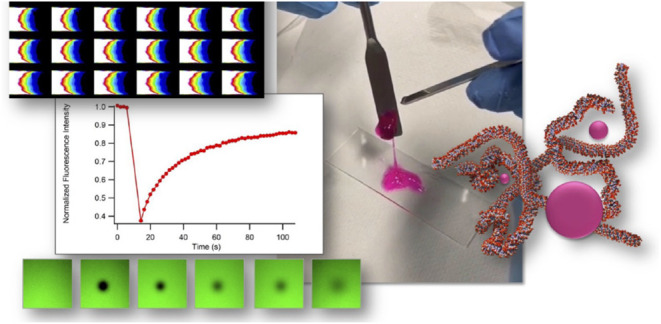

## Introduction

Supramolecular physical hydrogels are an attractive class of materials that could find applications in several biotechnological fields, such as controlled release of pharmaceutical molecules, tissue engineering, 3D bioprinting, and biosensor production.^1,2^ Indeed, the dynamic reversible nature of the weak interactions responsible for the formation of the entangled 3D *fishnet*-like structure endows them with self-assembling, self-healing, and shear thinning properties, combined with the ability to absorb a large amount of water.

Guanine hydrogels (G-hydrogels), produced by mixing Guanosine (Gua) and Guanosine 5′-monophosphate (GMP) in water, belong to this emergent class of smart materials.^3^ GMP is known for its hierarchical self-assembling in water when metal cations (as potassium) are present:^4^ GMP forms planar tetramers (G-quartets), stabilized by noncovalent Hoogsteen bonds, which stack one on the top of the other creating long helicoidal structures (G-quadruplexes, see Figure 1).^4^ When Gua is added to a GMP solution, a few GMP molecules are replaced in the forming G-quartets. The mixed planar tetramers are still able to stack, but G-quadruplexes are less charged, more flexible, and sticky. Depending on the amount of added Gua, a very stable hydrogel is formed, made from a 3D network of intertwined and knotted G-quadruplexes and capable of containing large amounts of water (up to 99%).^5^

**Figure 1:**
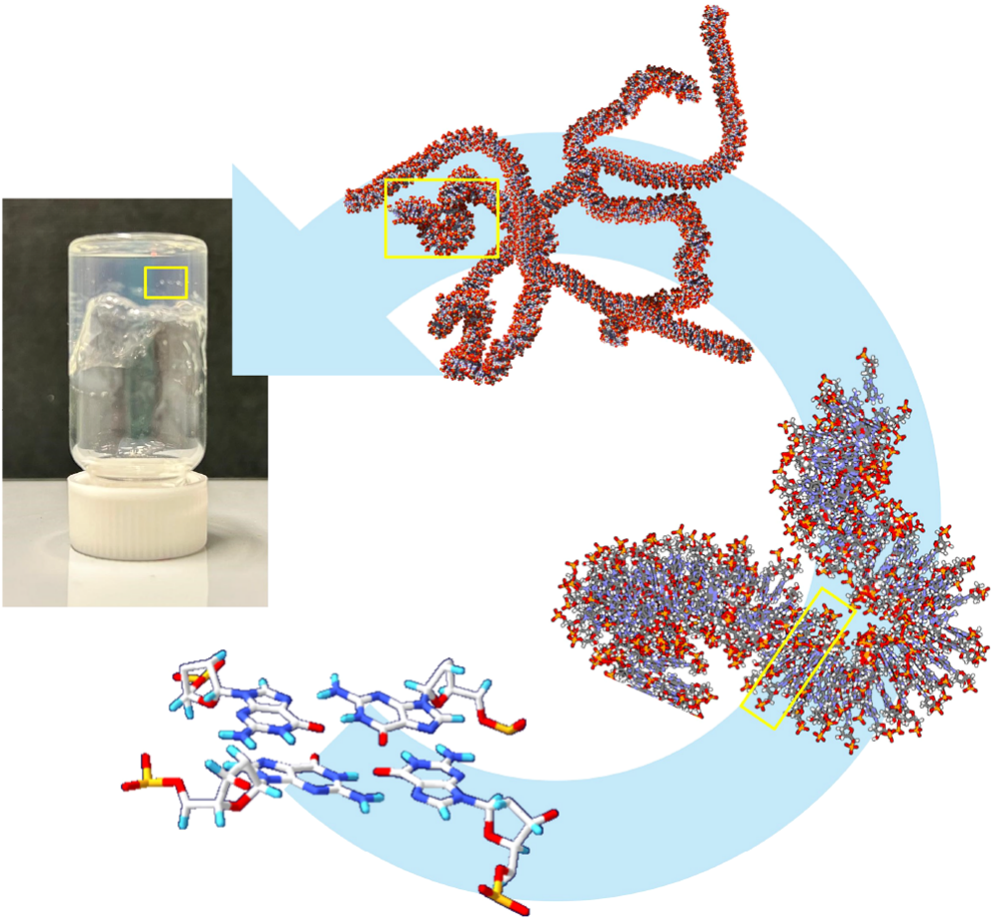
Hierarchical process of GMP and Gua self-assembly from a G-quartet to a flexible G-quadruplex and finally to the G-hydrogel. Following the arrow direction, the small yellow boxes highlight the different supramolecular aggregate.

There are some interesting features to stress: first, the metal cation is located in the central cavity of the G-quartet and forms a complex with oxygens of two staked tetramers, playing a pivotal role for the stabilization of the G-quadruplex.^6^ Second, the addition of Gua is essential for the formation of the hydrogel. In fact, the relative amount of Gua:GMP molar ratio controls the number of charges per G-quadruplex unit length and, in turn, the level of stiffness and intertwining of the G-quadruplexes and the number of physical cross-links inside the hydrogel, leading to a less or more entangled network, whose transport properties are likely to be different.

The objective of this paper is to assess by a multitechnique approach the transport features of the G-hydrogel as a function of the gel composition and of the properties of a few selected probes. According to the G-hydrogel structure, it is evident that pH changes and salt addition, as well as the presence of metal ions (such as Na^+^, which is known to differently stabilize the G-quadruplexes,^6^ or Mg^2+^, which determines the G-quadruplex condensation and precipitation^7,8^ ), can have a large effect both on the interaction of probes with the G-quadruplexes and on the transport properties of the matrix. However, pH and ionic strength (IS) are intrinsic properties of the G-hydrogel, governed by its composition and preparation method. Adding external buffers or salts could alter the gel native structure, introduce unwanted interactions, or complicate the interpretation of the diffusion results. Therefore, to reduce experimental complexity and focus on intrinsic material behavior, the G-hydrogels were prepared here at different Gua:GMP molar ratios and water content (e.g., without externally controlling pH or IS) and the probes were directly dissolved in the hydrogel water phase or in aqueous external solutions. However, PBS (phosphate-buffered saline) solutions were used for release experiments: 1× PBS was used to mimic physiological conditions in terms of ionic strength (IS ≈ 160 mM), pH (≈ 7 4), and osmolarity, while 10× PBS (pH ≈ 7 and IS ≈ 1.6 M) was considered to test eventual effects on drug solubility, diffusion, interaction, hydrogel swelling, and structure stability.

Four different fluorescent probes of different molecular weight and hydrodynamic radius, polarity, charge and, eventually, known to bind DNA (see Table 1), were considered (see Figure S1 in the Supporting Information, SI). As a result, unless the probes have a strong affinity for quadruplexes, the determined diffusion coefficients were observed to scale with the water content and the amount of GMP in the hydrogel, e.g., as a function of the mesh size. Similarly, for the same gel composition, the coefficients scale with the hydrodynamic radius. Diffusion coefficient properties were confirmed following probe penetration in the hydrogel during loading experiments. Finally, the dependence of the release time-constants on the diffusion coefficients confirmed that in 1× PBS (where the hydrogel structure does not undergo destabilization), the transport mechanism mainly follows a Fickian diffusion.^17,18^

**Table 1:** Properties of Fluorescent Probes: ^*a*^ Thioflavin T, ^*b*^4’,6-Diamidine-2-phenylindole, ^*c*^Rhodamine B, ^*d*^Fluorescein Isothiocyanate-Dextrans at Different Molecular Weight (MW)^a^

	polarity	charge	MW	*R*_H_	
name	(overall)	(pH ca. 8)	(Da)	(Å)	ref
ThT^*a*^	amphipathic	positive	318.9	∼5	^9–11^
DAPI^*b*^	amphipathic	positive	277.3	6–7	^12^
RhB^*c*^	hydrophilic	positive	479.0	7.8	^13^
FITC-dx 4^*d*^	hydrophilic	negative	∼4000	14.0	^14^
FITC-dx 10^*d*^	hydrophilic	negative	∼10000	29.0	^15^
FITC-dx 20^*d*^	hydrophilic	negative	∼20000	52.0	^16^
FITC-dx 70^*d*^	hydrophilic	negative	∼70000	72.0	^16^

a *R*_H_ indicates the hydrodynamic radius.

## Materials and Methods

### Materials

Guanosine 5′-monophosphate acid form (GMP-H^+^) (Santa Cruz Biotechnology, Dallas, Texas) was dissolved in distilled water at concentration of 16.5 mg/mL and titrated to pH 9 by using 1 M KOH. Pellets of GMP-K^+^ (GMP, from here) were then obtained by ethanol precipitation followed by two cycles of centrifugation at 4000 rpm for 15 min. The white, wet compound was freeze-dried by lyophilization overnight. Gua, DAPI, ThT, RhB, and FITC-dextran of 4 different molecular weights were purchased from Merck KGaA, (Darmstadt, Germany) and used as obtained.

### Hydrogel Preparation

Stock solutions of 200 mg/mL GMP and 150 mg/mL Gua were prepared in distilled water. G-hydrogels were prepared in Eppendorf tubes by mixing GMP and Gua solutions at the desired Gua:GMP molar ratio (from 1:4 to 1:1) and by adjusting the water content as required (90, 95, and 98%, v/v). In all cases, gel formation was immediate, but homogeneity was ensured by heating the mixtures up to 85 °C for 2–3 min, i.e., until a clear liquid solution was formed, and then allowing the samples to cool to room temperature. After 10 min of equilibration at 20 °C, the formed hydrogel was transparent and clear. Note that the quality of the gel formed is independent of the order of mixing of the two solutions.

The pH of the prepared hydrogels was approximately 8, while the calculated intrinsic IS ranged from 0.04 (1:1 98%) to 0.3 M (1:4 90%), as it depends on the amount of GMP (and K^+^) used in the sample preparation. Note that the correct charge balance is a prerequisite for hydrogel formation and stability,^3,5^ so that the addition of salts (e.g., KCl) to fix the ionic strength has been avoided.

### UV–vis Absorption Titration Experiments

UV–vis absorption spectra were measured using the DeNovix DS-11 spectrophotometer/fluorometer (DeNovix Inc., Wilmington, DE).

Dye titration with G-hydrogels prepared at different Gua:GMP molar ratios was performed by adding small aliquots of concentrated G-hydrogel (90% of water) to dye solutions prepared at concentrations of 7.2 × 10^–5^ M for DAPI, 7.8 × 10^–5^ M for ThT, 1.3 × 10^–5^ M for RhB, 7.5 × 10^–5^, and 1.1 × 10^–5^ M for 4k and 70k FITC-dextrans. The final volume of the mixtures was adjusted to 1 mL. The titration ranges from 0.001 to about 0.06 ligand-to-G4 molar ratio. During titration, the pH remained constant.

Some titration experiments were also performed at different total ionic strengths, controlled by adding KCl to the dye solution. The UV–vis absorption spectra of ThT for the sample in a 0.004 ligand-to-G4 molar ratio and at IS ranging from 0.40 to 0.64 M are shown in SI (Figure S2 of SI): the peak position and absorbance did not change with the ionic strength, at least within the considered range.

The interaction was described as a binding-diffusion process, characterized by a diffusion coefficient (*D*) and two binding rate constants (*k*_1_ and *k*_2_), from which the binding equilibrium constant *K*_b_ can be determined as *k*_1_/*k*_2_ ^19^ ratio. Indeed, eq 1 describes the conventional first-order binding process

$$F+S\overset{K_{b}}{⇄\,}\;B$$ 1

where F refers to free ligands in solution, S to the binding sites (here assumed to correspond to the G-quartet units present in the whole G-hydrogel), and B to the bound dye/G-quartet complexes.^20,21^

The stoichiometry and the intrinsic binding constants *K*_b_ were determined through the nonlinear regression method described by Thordarson et al.^22–24^ using the online supramolecular Bindfit software.^25^

### SAXS/WAXS Experiments

Small- and Wide-Angle X-ray Scattering (SAXS and WAXS) experiments were performed at the I22 beamline of the Diamond Light Source (Harwell, UK). The experiment exploited the mail-in service. The 3 M camera anisotropic SAXS/WAXS I22 setup and a Pilatus P3-2 M (silicon hybrid pixel detector, DECTRIS) detector with a pixel size of 172 μm^2^ were used. The final investigated *Q*-range (being *Q* as the modulus of the scattering vector, defined as 4πsinθ/λ, where 2θ is the scattering angle and λ the wavelength) was 0.4–3.0 nm^–1^ for SAXS and 3.0–50 nm^–1^ for WAXS. Hydrogels were measured in 1 mm thin-walled capillary tubes. In order to avoid radiation damage and to obtain reasonable statistics, SAXS/WAXS experiments were performed using short exposition times (30 s/frame) and considering different positions of the capillary in which the sample was held, for a total of 4–20 frames. The frames were then averaged. 2D data were corrected for background, detector efficiency, and sample transmission: I­(*Q*) *vs Q* curves were then obtained by the radial average of the 2D data.

### FRAP Experiments

FRAP experiments were performed by using a Nikon A1R+ laser scanning confocal microscope (Nikon, Japan). A combination of 405/488 nm lasers was used for G-hydrogel samples loaded with ThT and FITC-dextran, while 488/561 nm lasers were used for RhB. Each laser was used at the maximum of its intensity (100%). Experiments were performed using a 20× NA 0.7 Nikon Plan-Apo objective. The confocal pinhole was set at 1.2 Airy units, while the fluorescence intensity image size was fixed to 512 pixels × 512 pixels. Photobleaching was performed on a 25 μm circular region of interest (ROI) in radius (this is the *nominal radius* expressed as *R*_n_, see eq 2); thus, the bleached area size of the nominal region was almost 2 mm^2^. Measurement of the fluorescence intensity over time was carried out by using the NIS Elements AR software suite (Nikon). Data were compensated for observational photobleaching (using a square reference ROI far from the circular one) and analyzed in terms of fluorescence half-time of recovery (expressed as τ_1/2_, see eq 2). All experiments were performed at a constant temperature of 20 °C.

FRAP samples were prepared with the fluorescent probe already dissolved inside the G-hydrogel. A hydrogel drop was placed on a glass slide, covered by a coverslip of 24 × 24 mm^2^, and then gently wiped for removing excess sample. The sample thickness is estimated to be between 10 and 100 μm depending on the viscosity of the considered hydrogel. For each sample, 5 different points on the slide were explored, so that the final diffusion coefficient (*D*_c_) was determined from the average value in the whole data set. Four prebleaching images, corresponding to around 4 s, were averaged to normalize the postbleaching data. Subsequently, the bleaching phase covered 3 images for around 3 s. The postbleaching scanning time consisted of around 75 images, thus approximately 145 s.

The diffusion coefficient was calculated through eq 2, developed by Kang et al.^26^

2 $$D_{c}=\frac{R_{n}^{2}+R_{e}^{2}}{8\cdot \tau_{1/2}}$$

where *R*_n_ (in μm) is the nominal radius fixed by the operator, *R*_e_ (in μm) the experimental radius, and τ_1/2_ (in s) the fluorescence recovery half-time. *R*_e_ is extracted from the first bleaching window by eq 3 ^26^

3 $$f\left(x\right)=1-K\cdot e^{-x^{2}/R_{e}^{2}}$$

where *K* is the bleaching depth parameter, determined by taking into account that, most of the time, the bleached area does not correspond exactly with the nominal bleach radius.^26^ τ_1/2_ was finally derived by fitting data through eq 4

4 $$F\left(t\right)=F_{0}+\frac{F_{\infty }-F_{0}}{t+\tau_{1}/2}$$

where *F*_0_ is the fluorescence intensity at the beginning of the recovery phase and *F*_∞_ is the fluorescence intensity reached at the end of the recovery phase.^27^

### Solute Loading

The loading capacity of G-hydrogels was analyzed by placing the hydrogel prepared at the usual compositions (1:1, 1:2, 1:4 Gua:GMP molar ratios and 90, 95, 98% water) in contact with a aqueous solution containing 1 mg/mL of the fluorescent molecule to be loaded.

In order to reduce the hydrogel swelling, a glass capillary of 1 mm diameter, filled at one end with the G-hydrogel and at the other end with the probe solution, was used. The capillary was fixed on a glass slide to permit optical microscopy observations. Diffusivity was analyzed by using a Nikon A1R+ confocal laser scanning microscope with a 4× objective, taking advantage of the time-lapse option offered by the microscope. Each observation consisted of 30 min of continuous acquisition: during this time, the fluorescence at the position where the interface between the G-hydrogel and the probe solution is initially localized was followed. The image analysis was performed using *ImageJ* software.

### Solute Release

The probe release was studied considering hydrogels prepared at the usual composition (1:1, 1:2, 1:4 Gua:GMP molar ratios) at 95% water and containing the fluorescent probes at the concentration of 0.2 mg/mL (4.2 × 10^–4^ M). Aliquots of 0.5 mL of the prepared G-hydrogels were inserted in a vial tube, which was then placed inside a beaker filled with 60 mL of phosphate saline buffers (1× PBS and 10×). By sampling every 15 min the PBS solution by UV–vis spectroscopy, the cumulative amount of solute released from the G-hydrogels was obtained as a function of time.

## Results and Discussion

The transport behavior of G-hydrogels was analyzed, determining the diffusion- and binding-dependent mobility of different probes (both hydrophilic and hydrophobic). Three different aspects were considered: first, the structural properties of the G-hydrogels prepared in the presence of the different solutes; second, the binding of the different dyes to hydrogel immobile sites; and third, the solute mobility inside the hydrogel. These issues were considered separately, but a multiscale model for diffusion was used to compare the results for the controlled release of solutes.

### Structural Characterization of Loaded G-Hydrogels

The structural properties of the G-hydrogels containing the different dyes were analyzed by SAXS and WAXS techniques. A few profiles obtained from sample prepared in usual compositions (1:1, 1:2, 1:4 Gua:GMP molar ratios and 90 and 98% water) and containing the fluorescent probes at a concentration of 0.12 mM (e.g., from 0.001 to about 0.006 ligand-to-G4 molar ratio, depending on water content) are reported in Figure 2: scattering data are very similar to each other and identical to those already published for the empty hydrogel,^3,5^ suggesting that both the hydrogel structure and the G-quadruplex characteristics are not affected by the presence of different solutes. The Teixeira mass fractal model, which combines the fractal model with the scattering of a flexible worm-like cylinder,^28^ was used to fit the SAXS curves at 98% of water. With this model, flexible quadruplexes are considered to aggregate to form fractal-like clusters with a correlation length ξ, corresponding to the average cluster size, and a self-similarity dimension of *D*_f_. Fitted parameters, reported in Table 2, indicate that, within the instrumental resolution, no structural differences are detected with ThT or DAPI present in the hydrogel. G-quadruplexes remain very flexible (the length of the Kuhn segments is compared to the thickness of a single G-quartet) and their radius (underestimated with this model^5^) remains the same. Also the parameters related to the fractal-like clusters do not change after the addition of the probes. It is noteworthy that the new measurements confirm the previous data:^5^ the fit of the 1:1 hydrogel data yields a fractal dimension of 1.7 with cluster correlation sizes of only 20 Å, while the 1:2 and 1:4 hydrogels have a smaller fractal dimension of 1.2 and larger cluster sizes (≈75 Å for the 1:2 hydrogels and ≈125 Å for the 1:4 ones). Therefore, the fits show that the 1:1 hydrogel has a higher fractal dimension, which can indicate the presence of a tighter network compared to those of 1:2 and 1:4 hydrogels. Indeed, the smaller cluster sizes observed for the 1:1 sample confirm that there are more physical cross-links in the network, as expected, because of the reduced electrostatic repulsions among the G-quadruplex surfaces. This point could be important, as the increased steric hindrances imposed by the presence of more cross-links (e.g., the reduced mesh size) can limit the mobility inside the 1:1 hydrogel, resulting in a lower value for the diffusion coefficient of a probe compared to the other gels.

**Figure 2:**
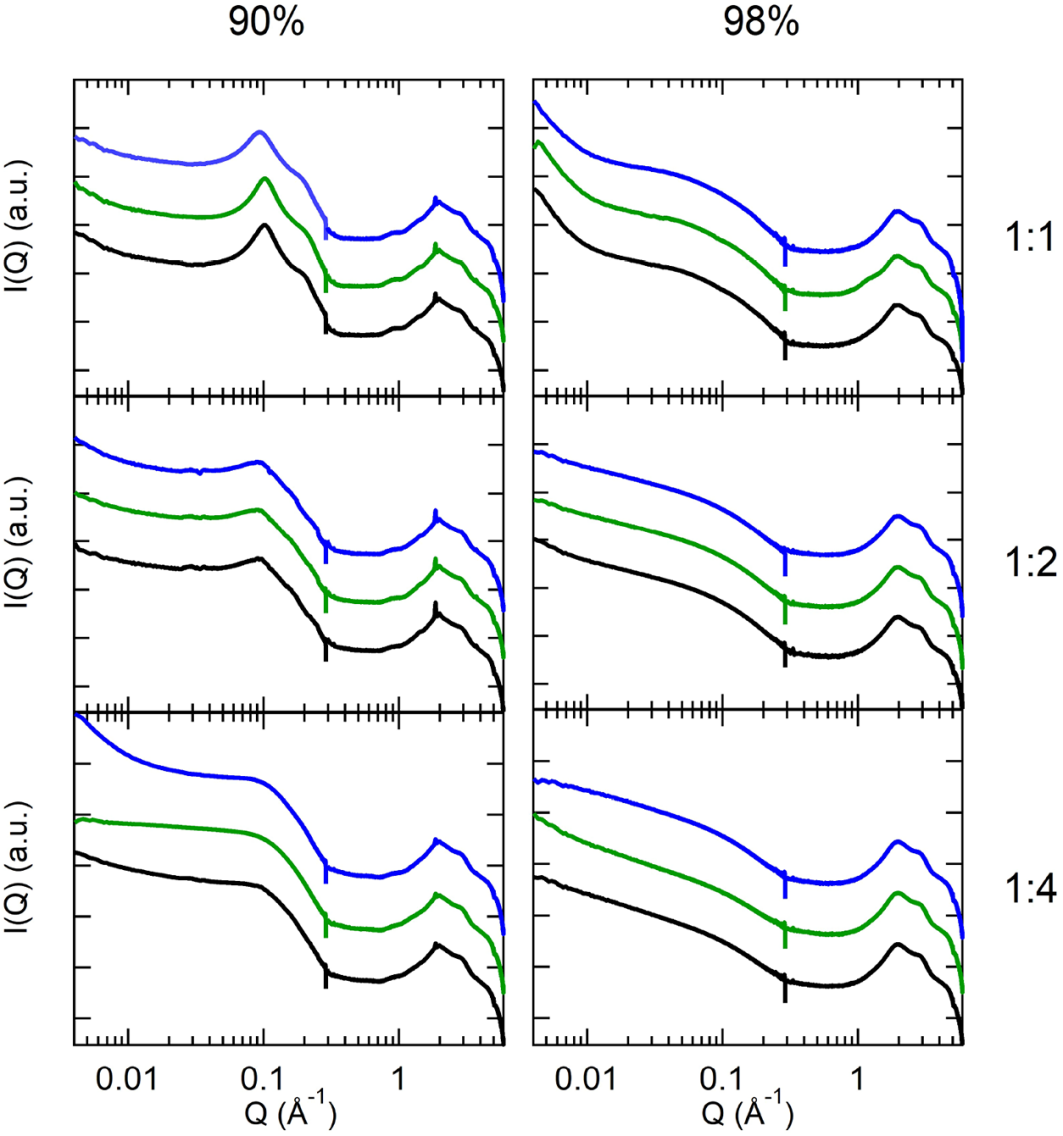
SAXS and WAXS data obtained from G-hydrogels at different water content and prepared at different Gua:GMP molar ratios, as indicated. Black curves refer to empty G-hydrogels (redrawn from Pepe et al.^5^), green curves to G-hydrogels loaded with ThT, and blue curves to those loaded with DAPI.

**Table 2:** SAXS Fitted Parameters for 1:1, 1:2, and 1:4 Hydrogels Prepared at 90 and 98% of Water (v/v), in the Absence and in the Presence of ThT and DAPI^a^

			1:1			1:2			1:4	
	error	empty^5^	ThT	DAPI	empty^5^	ThT	DAPI	empty^5^	ThT	DAPI
90%										
*R* (Å)	±0.5	12.5	12.6	12.5	13.3	13.2	13.8	13.1	12.7	13.1
*h* (Å)	10%	123	120	136	113	111	101	98	109	90
*U*_0_ (*kT*)		0.10	0.11	0.07	0.02	0.01	0.31	0.10	0.03	0.04
σ (Å)		2.55	2.59	2.87	3.79	1.84	1.34	2.70	2.71	2.32
98%										
*D*_f_	±0.2	1.7	1.7	1.7	1.2	1.2	1.2	1.2	1.2	1.1
ξ (Å)		23.0	22.8	23.1	73.4	76.4	70.4	122.0	130.2	127.5
*b* (Å)	±1.5	4.6	4.2	5.0	5.2	4.5	4.8	3.1	4.9	4.3
*L* (Å)		79.1	57.9	62.2	108.7	110.0	112.1	108.5	100.5	86.4
*R* (Å)	±2.5	8.2	9.2	9.1	8.6	9.0	8.4	8.9	7.8	9.5

a For samples at 90% of water, the hard sphere plus square well potential model for cylindrical objects was used:^29,30^ *R* and *h* are the cylinder radius and height, while *U*_0_ and σ the potential depth and width. For samples at 98% water, the Teixeira Mass Fractal Model was used:^28^ *D*_f_ is the fractal dimension, ξ the correlation length, *b* the Kuhn length, *L* the contour length, and *R* the cylinder radius. Where not indicated, errors are on the order of 20%.

For samples at 90% of water, SAXS profiles were analyzed by considering a hard sphere plus square well potential model for cylindrical objects.^5,29,30^ As before, the fitted parameters (see Table 2) are very similar to each other, confirming that ThT and DAPI do not determine the structural effects visible under these conditions. Among others, the square well potential parameters confirm the presence of the attractive potential, which is related to the overall charge reduction due to the replacement of a certain number of GMP molecules along the quadruplexes with the electrically neutral Gua, and which contributes to establishing tighter contacts between the aggregates. According to these data, the gel stability is not affected by the addition of ThT and DAPI.

Concerning WAXS profiles, the analysis focused on the narrow Bragg peak observed at about *Q*_s_ = 1.85 Å^–1^, as this peak is related to the G-quartet stacking distance and is then indicative of the formation of G-quadruplexes. As shown in Figure 3, no changes in the stacking distance (calculated as 2π/*Q*_s_) were detected after the addition of ThT or DAPI, suggesting that ligands bind to the external surface of the quadruplex, at least at the analyzed concentrations.

**Figure 3:**
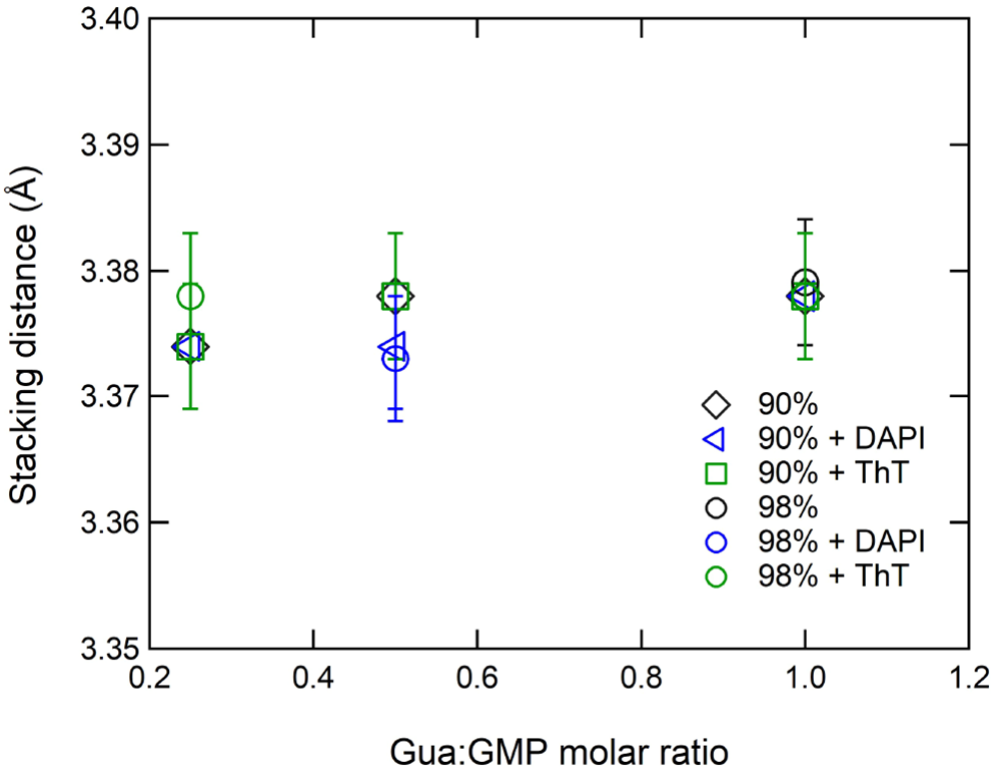
G-quadruplex stacking distances measured from G- hydrogels prepared at different water contents and at different Gua:GMP molar ratios loaded with ThT or DAPI.

The stability of the gel and the structure of the quadruplexes do not seem to depend on the presence of ThT or DAPI, but it is evident that the concentration of the probes was very low. However, a series of SAXS/WAXS experiments were performed at larger probe concentrations (up to 1.0 mM, e.g., up to a ligand-to-G4 molar ratio of 0.06, which corresponds to the maximum concentration used in the titration experiments). The obtained results (see the case of 1:4 98% hydrogel in Figure S3 of SI) confirmed the absence of difference between the experimental profiles.

### Binding of Dyes to G-Quadruplexes: Binding Constants

In order to study the diffusion properties of the different probes in the hydrogel, their binding capacities with the quadruplexes were first investigated. Noncovalent interactions were assessed by UV–vis titration experiments, as all ligands show absorption bands in a UV–vis region clearly distinguishable from those related to the guanosine supramolecular aggregates. Therefore, the expected changes in absorption between the ligand free in solution and the one complexed with the G-quadruplex can be used to obtain the association constant.^31^

The titration absorption spectra and the corresponding main changes observed as a function of the quadruplex concentration are shown in SI (Figures S4 and S5, respectively). Different behaviors can be recognized. For DAPI and ThT, results indicate strong interactions: during titration, a clear red-shift from 345 to 365 nm and from 415 to 435 nm is detected for DAPI and ThT, respectively, but a strong hypochromic effect is observed only for DAPI. As shown in Figure S5 of SI, changes of both absorption intensity and position appear independent of the Gua:GMP molar ratio (at least, within the experimental errors). No significant changes are indeed observed in the case of the hydrophilic probes, except for a minor increase in absorbance detected in the case of RhB. Therefore, no interactions occur for FICT-dextrans, while weak electrostatic bindings to quadruplexes are probably established for RhB.

To avoid possible problems related to the validity and reliability of Benesi–Hildebrand approach,^22^ data were then analyzed by using the Bindfit v0.5 program.^25^ The best fit results, obtained considering a 1:1 stoichiometry, are shown in Figure 4, while binding constants are reported in Table 3.

**Figure 4:**
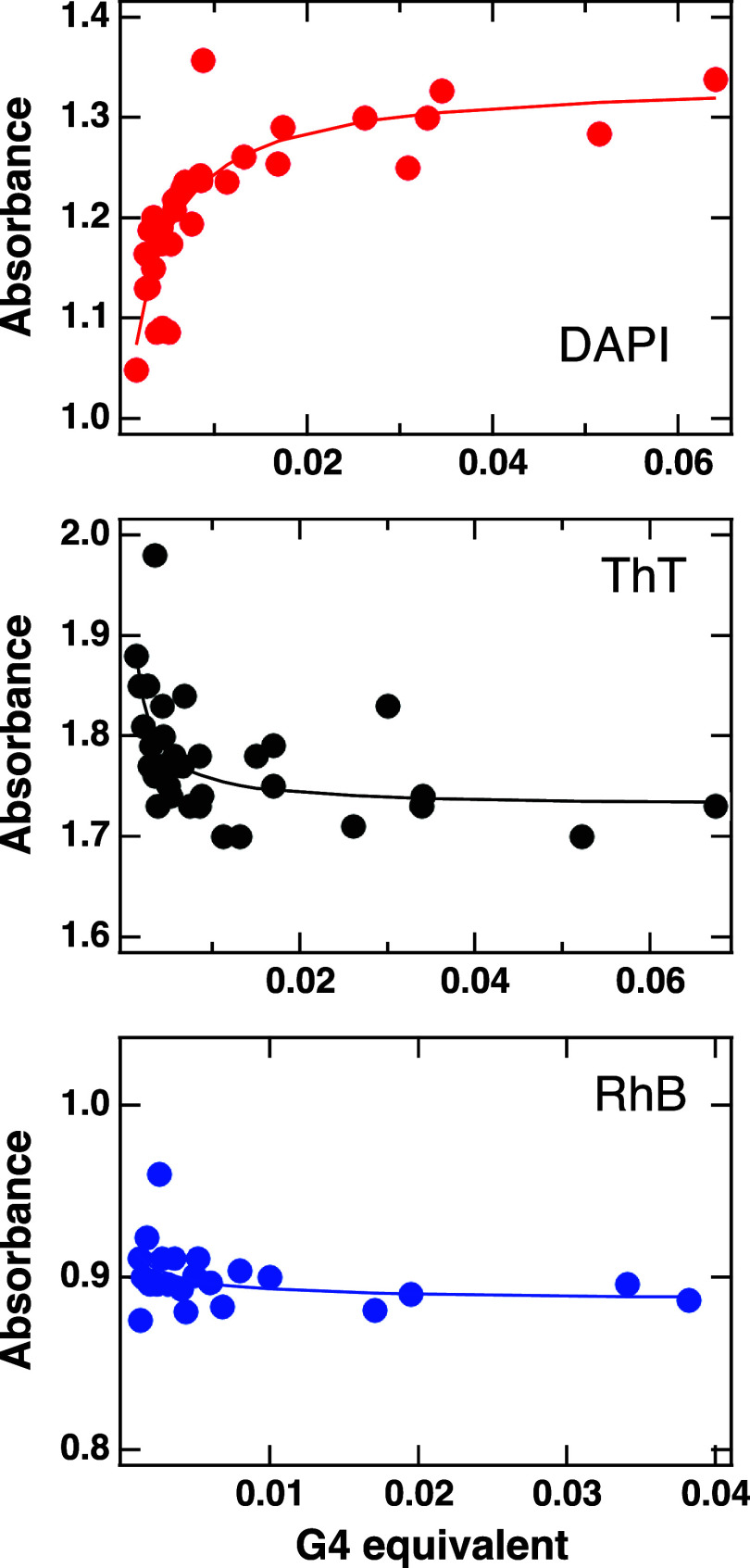
Bindfit plots relative to G-quadruplexes in the presence of DAPI, ThT, and RhB. Note that the guest/host equivalent ratio is calculated as the ratio between the molarity of the ligand and that of G-quartets (obtained by dividing the sum of Gua and GMP molarities by a factor of 4).

**Table 3:** Binding Constants Obtained by G-Hydrogel Titration for the Three Different Probes

ligand	*K*_b_ (M^–1^)	error (%)	Δ*G* (kJ mol^–1^)
DAPI	5.7 × 10^4^	21	27.3
ThT	8.0 × 10^3^	36	22.4
RhB	1.4 × 10^2^	46	12.3

Data are scattered and errors are quite large, but at least two points can be noticed: first, *K*_b_ for DAPI is larger than the binding constant observed for ThT, suggesting different interaction mechanisms.^32^ Second, the binding constant for RhB is very low. Unlike DAPI and ThT, RhB is polar (hydrophilic), and its binding modes are likely different and less effective.

It is important to note that the description of the mechanics that governs this kind of interaction is behind the scope of this work, but tentative comments can be presented, in particular by referring to the modalities described for double-helical DNA or in noncanonical nucleic acid conformations.^12,33,34^ In DNA, two binding modes were described for DAPI:^12^ minor groove binding, which saturates at low DAPI concentration and presents equilibrium binding constants of the order of 10^7^ M^–1^, and intercalation, occurring only after the saturation of the first mode and presenting a moderate affinity (the equilibrium binding constant is ≈10^5^ M^–1^). Both binding modes are affected by the buffer conditions (e.g., ionic strength). For ThT, the binding to DNA has been described according to three modes: specific binding to DNA cavities, intercalation between DNA bases, and external binding to DNA phosphate groups.^35^ The binding of ThT to telomeric G-quadruplexes has already been reported,^33,36,37^ providing insights into a probable binding mode where the ThT molecule occupies the top G-quartet of the telomeric structure or interacts with guanosines via end-stacking. Significant binding constants of about 10^5^–10^6^ M^–1^ were reported.

In the G-quadruplexes described here, the backbone is absent, and DAPI or ThT intercalation would have required the release of the cation strongly complexed between two superposed G-quartets without quadruplex breaking. SAXS and WAXS results show that the G-quadruplex length and G-quartet stacking distance are independent of the presence of DAPI or ThT, suggesting that intercalation should not occur. Moreover, no effects were detected by adding KCl to the solutions, suggesting that interactions are dominated by the G-quadruplex hydrophobicity.

Indeed, it would seem much more likely that the G-quadruplex surface provides a stable binding groove site for both DAPI and ThT.^31,33^ However, binding constants are smaller than those found in DNA or in telomeric quadruplex binding, probably confirming the strong sensitivity of the two probes to the surface topology.^33^ In any case, DAPI can be considered more hydrophobic than ThT, and then probably tends to partition better into hydrophobic regions, like the groove of G-quadruplex grooves.

The binding of RhB to DNA has been also already studied^34^ and a groove binding mode has been reported. The interaction has been described to include both electrostatic and nonelectrostatic contributions, but affinity resulted rater low, in the range of 10^3^ M^–1^. Our results are in good agreement with these observations: with respect to DAPI and ThT, RhB shows a lower binding affinity to G-quadruplexes. Moreover, as suggested above by considering the different surface characteristics, the binding constant is smaller than that observed with DNA.

### Diffusion in G-Hydrogel: FRAP Experiments

To provide evidence of the diffusivity properties of G-hydrogels, FRAP experiments were performed as a function of both gel composition (1:1, 1:2, and 1:4 Gua:GMP ratios) and water concentration (90, 95, and 98% v/v). The diffusion coefficients *D*_c_ were determined as indicated above, and the results are reported in the form of histograms in Figure 5. For sake of clarity, the *D*_c_ dependence on the molecular weight of the different probes is shown in SI (Figure S6): as a general rule, lower MW probes show higher diffusivity. Two points should be noticed: first, ThT coefficients differ from those derived for the other probes (they are rather small compared to the MW); second, no fluorescence recovery was observed in the case of G-hydrogels loaded with DAPI. The binding affinity is probably so high, e.g., attractive interaction so strong, that the ligand is practically immobile.

**Figure 5:**
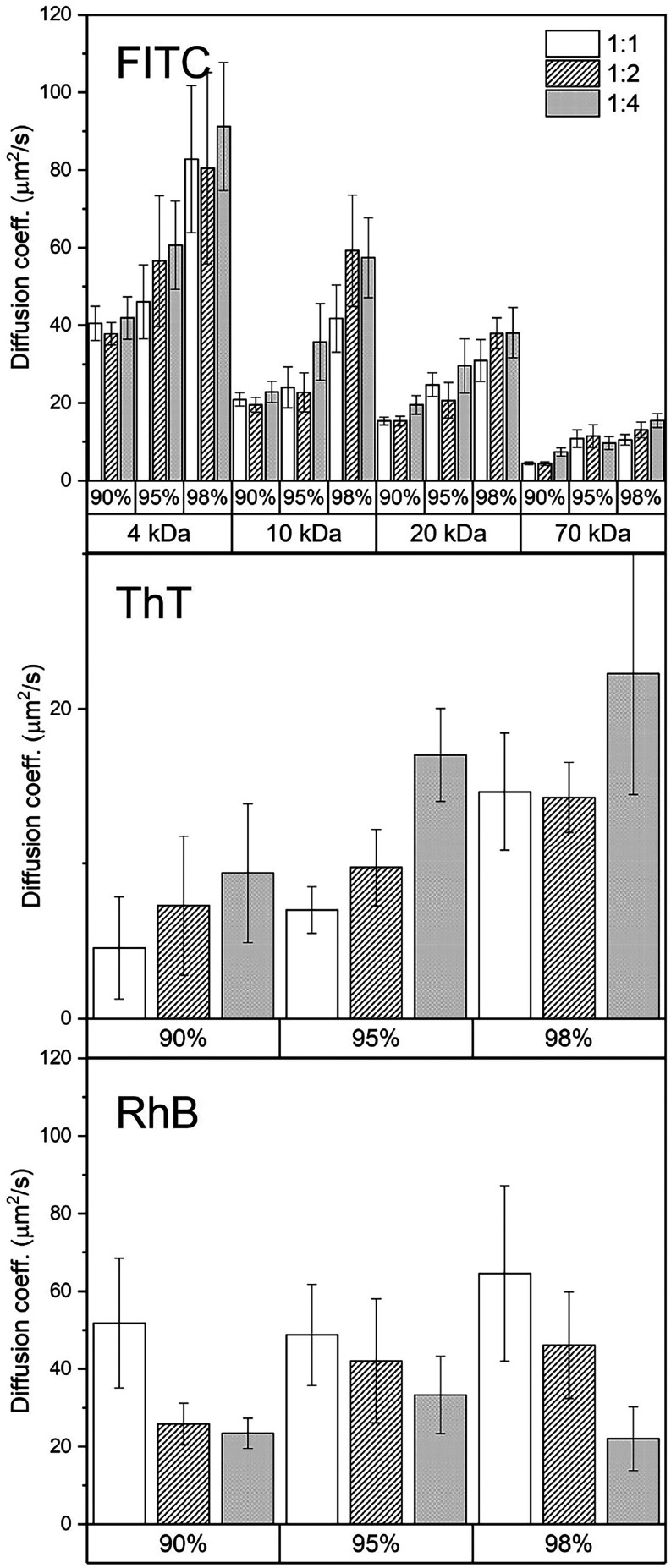
Overview of the diffusion coefficients *D*_c_ observed for FITC-dextrans at different molecular weights (top), ThT (middle), and RhB (bottom) in G-hydrogels prepared at different Gua:GMP molar ratios (1:1, 1:2, and 1:4) and water contents (90, 95, and 98% v/v).

FRAP revealed two different situations: low diffusion coefficients were obtained for ThT, in full agreement with the large binding constant revealed by UV titration experiments, while large diffusivity was observed for FITC-dextrans and RhB, probably because of their strong hydrophilic nature. However, diffusion coefficients show a clear dependence on the G-hydrogel composition, with notable differences between the three samples: *D*_c_ increases as a function of both water content and Gua:GMP ratio, but the dependence is minimal for ThT and RhB, whereas it is considerably high and closely related to the molecular weight of the probes for FITC-dextran.

It should be noted that the diffusivity of a solute with hydrodynamic radius *R*_*h*_ in a pure liquid is given by hydrodynamic theory, which describes an inverse relationship between the diffusion coefficient and the hydrodynamic radius of the diffusing particles. Nevertheless, the solute diffusivity in a hydrogel is altered by the presence of the 3D network, which represents a barrier for its diffusion within the liquid (obstruction theory). In particular, the aqueous part of the hydrogel occupies a continuous porous region of nanometric cross-section that extends between the gel strands and shows a characteristic average size ξ (referred to as mesh size), which corresponds to the correlation length of the network. In this case, the mesh properties are influenced by the structural characteristics of the filaments, i.e., by the number of charges per unit length, which, in turn, controls the G-quadruplex flexibility, entanglement, cross-linking, and swelling ability. Therefore, the hydrogel composition and water content are expected to influence its transport properties

The first panel of Figure 5 shows the results obtained for FITC-dextrans. For all hydrogel compositions, *D*_c_ decreases as the molecular weight of FITC-dextran increases, in full agreement with the Stokes–Einstein equation. Moreover, the rate of diffusion also depends on the hydrogel composition and increases as a function of both the amount of water inside the G-hydrogel and the Gua:GMP molar ratio. It has been stated that steric interactions (solute particles can travel long distances through a hydrogel if they are small enough to seep through the voids between the polymeric chains) and hydrophobic and electrostatic forces between the solute particles and the polymers control the diffusion properties inside the hydrogel.^38^ Then, in the absence of electrostatic interactions between the 3D net and the diffusing FITC-dextran molecules, as suggested by UV–vis titration experiments, the observed effects can be strictly related to the structural and viscoelastic properties of the hydrogel. The role of steric obstruction is very clear: first, the hydrogel swelling causes a relaxation of the 3D net, and therefore, an increase of the mesh size should be observed on dilution (see the simple net model in SI, Figure S7). Diffusing molecules move more easily and faster at 98% of water than at 90%. Second, the increase in quadruplex-to-quadruplex repulsive forces when the GMP content increases gives rise to a stronger swelling network, in which the less flexible G-quadruplexes are scarcely entangled and rather far from each other. Namely, the structure becomes more rigid, but the quadruplex-to-quadruplex repulsive forces induce an increase of the average mesh size.^3,5^ Again, the diffusion of FITC-dextrans through the hydrogel richer in GMP is easier. Concerning the viscoelasticity, it is important to consider that the hydrogel is a complex two-phase system. As indicated above, one phase represents a relatively sparse spatial 3D network of quadruplexes, while the second one is the water region, which occupies the micro- and nanoporous space between the quadruplex net. Consequently, the eventual dependence of the viscosity of the water phase on the hydrogel composition and entanglement should be taken into account.

FRAP results for ThT are reported in the second panel of Figure 5. Even if the dye MW is rather small (319 Da), the *D*_c_ values are surprisingly low and only slightly dependent on the hydrogel composition. In this case, UV–vis titration results suggested that hydrophobicity and electrostatic effects play a key role in the binding of ThT to G-quadruplexes: the binding slows down the probe diffusion. Regardless, the properties of the hydrogel regulate ThT diffusion exactly as they controlled diffusion for FITC-dextrans: *D*_c_ values increase with a higher water content in the G-hydrogel and a higher Gua:GMP molar ratio.

*D*_c_ coefficients for RhB, reported in the third panel of Figure 5, indicate a fast diffusion in the G-hydrogel, in good agreement with its low molecular weight. However, no dependence on the amount of water is observed, whereas the dependence on the Gua:GMP molar ratio is opposite than that observed for FITC-dextrans. UV–vis titration experiments showed a moderate binding to G-quadruplex groove, probably mediated by electrostatic interactions.^34^ Therefore, diffusion coefficients are probably reduced when the number of charges per unit length in the G-quadruplexes (e.g., the GMP content) increases due to the more favored groove binding.

To derive the transport properties of the G-hydrogel, results were analyzed considering the recently published multiscale model for solute diffusion (MSDM),^39,40^ which combines the three main theories for the diffusion of solutes in hydrogels: (i) the friction between the solute and the surrounding hydrogel matrix (hydrodynamic theory); (ii) the solute translation via dynamic empty volumes between molecules (free volume theory); and (iii) the solute transport against the hydrogel 3D net barrier (obstruction theory). Within MSDM, all of these theories are combined: the solute diffusivity, expressed as the *D*_c_/*D*_0_ ratio, whit *D*_0_ diffusion coefficient of the particle solute in pure water, is described by the probability of diffusing via free volume voids and along the aqueous solution through a mesh of size ξ, as derived from Fick’s law^40^

5 $$D_{c}/D_{0}=\mathrm{erf}\left(\frac{r_{\mathrm{FV}}}{R_{h}}\right)\exp\left(-\frac{\phi_{p}}{1-\phi_{p}}{\left(\frac{R_{h}}{r_{\mathrm{FVW}}}\right)}^{3}\right)+\mathrm{erfc}\left(\frac{r_{\mathrm{FV}}}{R_{h}}\right)\exp\left(-\pi {\left(\frac{R_{h}+R_{f}}{\xi +2R_{f}}\right)}^{2}\right)$$

where ϕ_p_ is the hydrogel volume fraction, *R*_h_ the hydrodynamic radius of the probe, *R*_f_ the cross-sectional radius of the strands forming the network (e.g., the G-quadruplexes), *r*_FV_ and *r*_FVW_ the average radii of the free volume voids (*r*_FVW_ in the case of water). According to the weighting factors (the error function and its complement), eq 5 indicates that the diffusion via free volumes dominates when the hydrodynamic radius of the solute is comparable to the average radius of the free volume voids (*R*_h_ ≅ *R*_FV_), whereas the mesh size becomes the limiting factor when the solute size is much larger than the free volume voids (*R*_h_ ≫ *R*_*F*V_). The solute diffusivities via free volume holes and through the mesh occur at distinct length scales, and thus, the probability for a solute to simultaneously diffuse by both mechanisms is essentially zero. In addition, intermolecular forces are considered negligible.

It should be evident that eq 5 captures only the hydrodynamic radius of the probe and cannot be applied when probe/hydrogel interactions are a major determinant of diffusivity. Therefore, the MSDM model was used only to analyze the diffusivity data of RhB and FITC. Normalized diffusivity obtained from hydrophilic noninteracting probes (FITC-dextrans and RhB) is reported as a function of the hydrodynamic radius in Figure 6. Best fit curves obtained by eq 5 are also shown, whereas the fitted parameters are presented in Table 4. Even if the data are very noisy and scattered, the fit is able to capture the experimental observations, including both the small diffusivity observed for RhB and the presence of a maximum as the FITC-dextran *R*_h_ increases (see Figure 5). In particular, the minimum suggests a low probability of diffusion through dynamic free volume voids due to the comparable size of RhB to *R*_FV_ (see Table 4), while the maximum in diffusivity is observed where the size of the FITC-dextrans is compared with the G-hydrogel mesh size.

**Figure 6:**
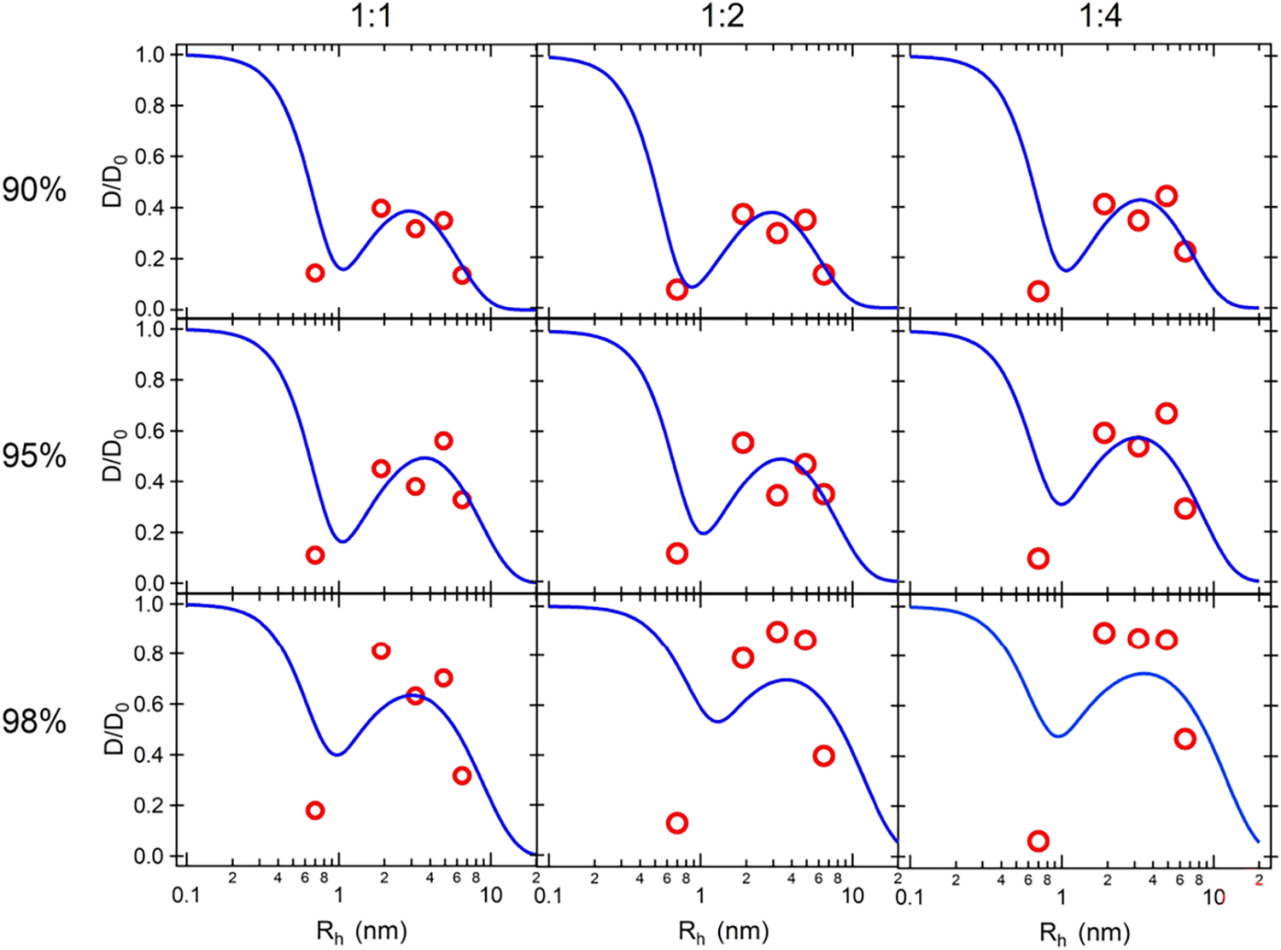
Fits by MSDM to experimental *D*_c_/*D*_0_ data for all of the investigated G-hydrogels loaded with hydrophilic probes.

**Table 4:** Parameters Obtained by Fitting *D*_c_/*D*_0_ Data with the MSDM Model^a^

water %	ϕ_p_	*r*_FV_, 1:1	ξ, 1:1	*r*_FV_, 1:2	ξ, 1:2	*r*_FV_, 1:4	ξ, 1:4
90	0.10	1.1 ± 0.5	8.7 ± 2.5	1.1 ± 0.3	8.6 ± 1.3	1.2 ± 0.6	10.6 ± 3.8
95	0.05	1.1 ± 0.5	12.0 ± 4.7	1.0 ± 0.5	13.1 ± 4.4	0.7 ± 0.4	13.2 ± 5.1
98	0.02	0.6 ± 0.4	14.3 ± 5.8	0.6 ± 0.4	19.6 ± 8.3	0.5 ± 0.3	20.0 ± 9.7

a Probe *R*_h_ values are reported in Table 1, *R*_f_ has been fixed to 1.3 nm which corresponds to the G-quadruplex radius^3^ (e.g., no multiple strands were considered to form the 3D network) and *r*_FVW_ was fixed to 0.27 nm.^40^ ϕ_p_ values are calculated from the hydrogel composition. Results are in nm.

Concerning the fitted parameters, the mesh size values (ξ) are in very good qualitative agreement with experimental observations: mesh size increases with water content due to the swelling of the net structure induced by the addition of water and when the repulsive interactions between G-quadruplexes increase (e.g., when the relative amount of GMP in the hydrogel increases). From a quantitative point of view, a comparison can be made considering SAXS results at 90% water (see Figure 2): the position of the correlation peak (from 6.0 to 7.4 nm, when the Gua:GMP ratio moves from 1:1 to 1:4 ^5^), and the correlation length derived by best fit analysis (from 2.8 to 11.4 nm^5^) reflect the mesh size of the hydrogel network. SAXS results and parameters reported in Table 4 are rather similar (within an order of magnitude, but errors on ξ are very high, probably because of the mesh inhomogeneity) and show a comparable dependence on the G-hydrogel composition.

The other fitted parameter is the average size of the empty voids between all molecules forming the hydrogel structure, *r*_FV_. The fit values are rather large compared to those reported in the literature,^40^ but it is likely that the free volume holes in G-hydrogels deviate from those in water due to the hydrophilicity of the G-quadruplex surfaces (free volume holes are larger as the water content is smaller).

These results demonstrate once again that G-hydrogels are tunable materials, and their properties can be perfectly tailored: by modulating Gua:GMP molar ratio and water content, hydrogels are able to effectively control the release of a drug or the movement of nutrients through a 3D scaffold for cell cultures.

### Solute Loading and Release

With reference to the transport properties of G-hydrogels, aspects related to the loading and release of a solute from the hydrogel were also investigated. This point is rather interesting, as the process is activated by placing the gel in contact with a aqueous solution. In such conditions, hydrogel swelling (possibly until degradation) and solute diffusion are expected to occur at the same time at the interface between the hydrogel and the solution.

Since these are the first loading and release experiments from G-hydrogels, three main aspects were fixed. First, in the loading experiment, no pH or IS control was performed: the gel was prepared in the usual way and placed in contact with an aqueous solution with the probe. Second, release experiments were performed against both 1× and 10× PBS solutions, in order to mimic physiological conditions and to test eventual effects due to hydrogel swelling, erosion, or relaxation induced by IS or pH changes. Third, with these premises, loading and release results must be considered with great attention and caution.

#### Solute Loading in G-Hydrogels: Assessment Inside a Glass Capillary

To reduce the convective flow and swelling of the hydrogel, which can affect the permeability of a substance, loading experiments were performed using a glass capillary filled with the G-hydrogel in close contact with an aqueous solution containing the fluorescent probe. In particular, FITC-dextrans loading on G-hydrogels prepared at the usual Gua:GMP molar ratios and water content was followed by fluorescence microscopy observations.

The temporal evolution of fluorescence observed at the hydrogel/dye interface demonstrates that the fluorescent probe propagates inside the gel. Examples for 1:4 98% G-hydrogel in contact with a FITC-dextran 4 kDa solution are reported in SI, Figure S8. In order to derive the kinetics of the loading process, the fluorescence propagation at each time (e.g., in each frame) was analyzed by measuring from left to right (e.g., from the probe solution to the G-hydrogel) the fluorescence intensity in a line parallel to the capillary and passing through its center. Data were then analyzed as a function of the distance from the solution/G-quadruplex interface, as indicated in SI, Figure S8.

Since the fluorescence dependence on distance from the interface is related to the penetration of FITC-dextrans inside the G-hydrogel, diffusivity coefficients (μm^2^/s) were derived by fitting experimental data to the following equation

6 $$F\left(x,t\right)\propto \mathrm{erfc}\left(\frac{x}{2\sqrt{D_{\mathrm{eff}}t}}\right)$$

where erfc is the complementary error function, *F* the fluorescence normalized to the initial time point (dimensionless), *x* the distance from the fluorescence probe reservoir (mm), *t* the time since the probe-hydrogel contact (s), and *D*_eff_ the effective diffusion coefficient (reported in μm^2^/s to have a comparison with the data from FRAP ones).^41,42^ The fitting curves are reported in SI, Figure S8, while the calculated diffusion coefficients for each combination of G-hydrogels and FITC-dextrans in form of histograms are shown in Figure 7.

**Figure 7:**
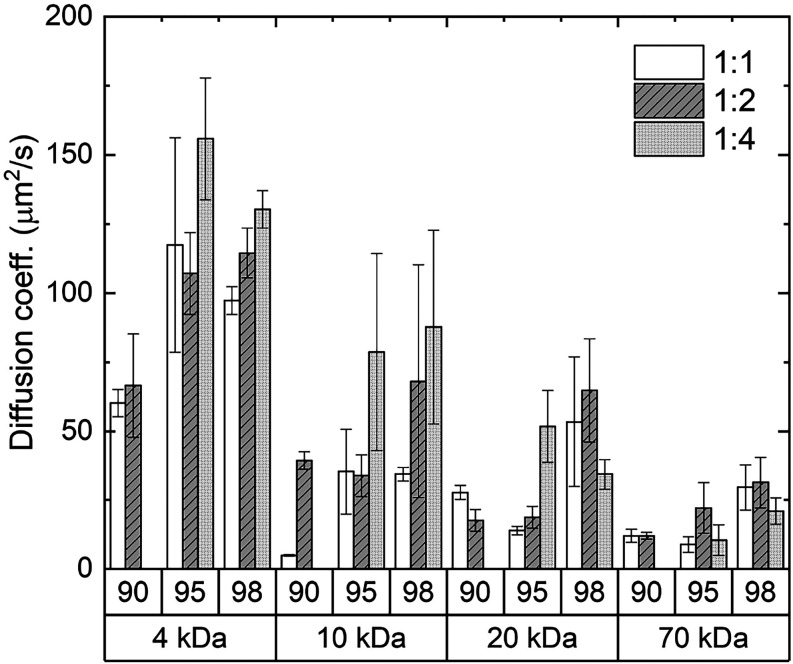
Diffusion coefficients (μm^2^/s) determined for loading of FITC-dextrans at different molecular weight (4, 10, 20, and 70 kDa) in G-hydrogels. As indicated, 1:1, 1:2, and 1:4 Gua:GMP hydrogels prepared at 90, 95, and 98% of water content were considered. No results for 1:4 90% sample are shown because of its high flow-ability.

Diffusivity values for probe loading are in full agreement with diffusion data determined by FRAP: *D*_eff_ increases by increasing the network charge (from 1:1 to 1:4 in terms of the Gua:GMP molar ratio) and the water amount; moreover, *D*_eff_ decreases when the dextran size increases, as expected. The striking similarity between the data obtained by FRAP and by capillary diffusion measurements deserves two comments: the first is related to the indication that the penetration of water at the interface is negligible, at least for the first hours. The second concerns the mechanism: in the absence of quadruplex-solute interactions, loading and diffusion in the hydrogel are primarily controlled by the mesh size. In the present case, the hydrogel composition (e.g., the correct charge balance) continues to have the utmost importance in determining the permeability properties of the G-hydrogel.

#### Solute Release from G-Hydrogels: Evaluation in Excess Solution

The release of loaded solutes from the G-hydrogel was then considered to demonstrate that a sustained drug release can be achieved with the G-hydrogel. Indeed, the experiment is complicated as self-assembled physical hydrogels can undergo transitional changes in response to environmental triggers: as reported above, the G-hydrogel can swell, shrink, degrade, or exhibit a sol–gel phase transition upon changes in conditions (composition, water content, salt, pH, cations, temperature). Since diffusion through the hydrogel network depends, among other things, on the mesh size, any effect that causes changes in the mesh size affects probe release. To take this point into account, release experiments were done against both 1× and 10× PBS solutions (pH ≈7.4 and IS 160 mM and pH ≈7 and IS 1.72 M, respectively).

Release results are shown in Figure 8: in particular, the fraction of probe released from a G-hydrogel to the solution at time *t* and infinite time, *M*_*t*_/*M*_inf_, e.g., the cumulative release fraction, is reported as a function of time.

**Figure 8:**
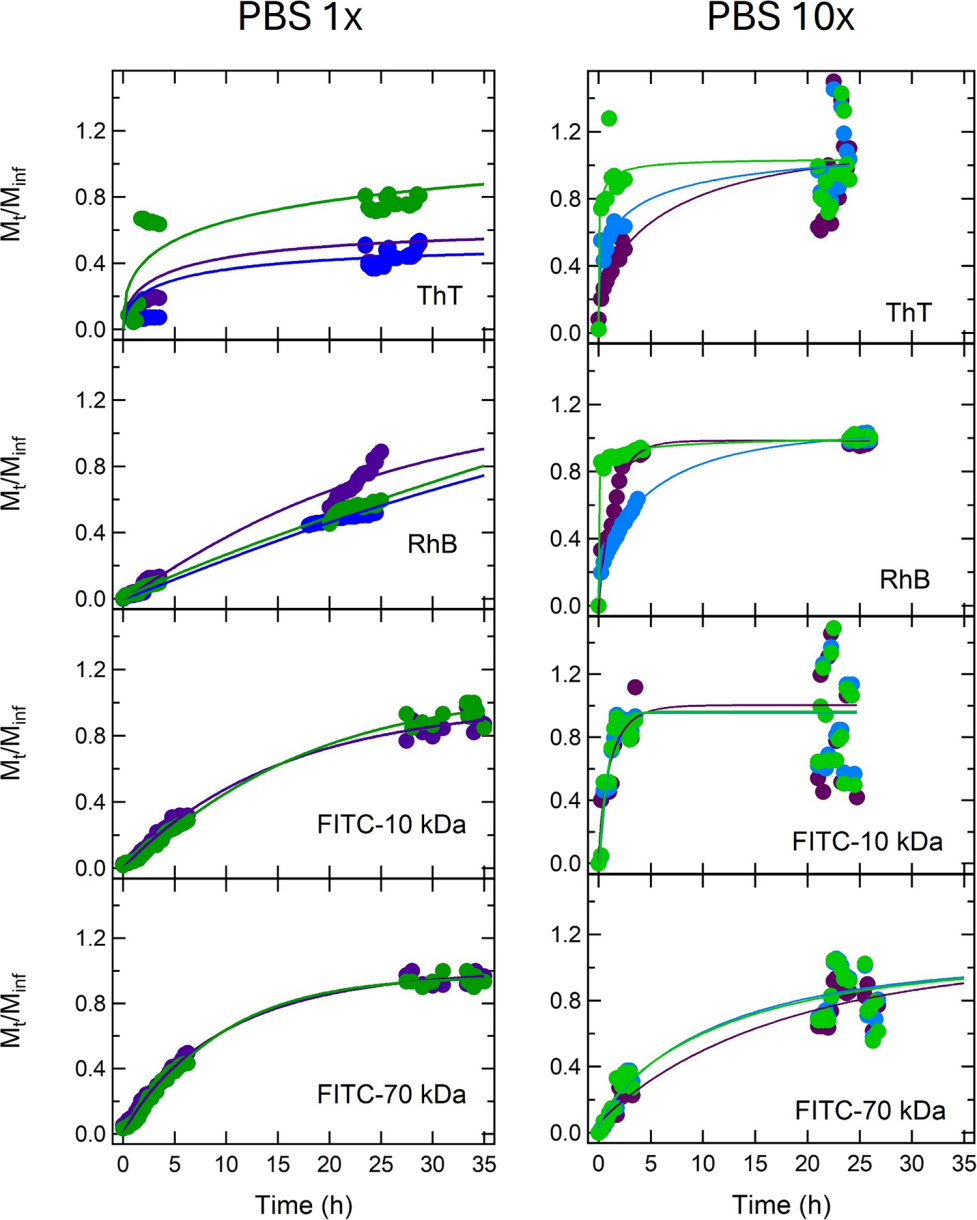
Time dependence of the normalized amount of ThT, RhB, FITC-dextran 10 kDa, and FITC-dextran 70 kDa released from G-hydrogels prepared at 95% water and at different Gua:GMP molar ratios to 1× and 10× PBS solutions. Purple, cyan, and green dots represent the probe release in 1:1, 1:2, and 1:4 G-hydrogels, respectively. Lines are best fits to the data obtained using the Weibull function.^43^

The amount of the released probe increases as a function of time according to a power-law, with release rates that depend on the considered compound, on the G-hydrogel composition, and on the external solution used for the experiment. To describe the kinetics of drug release and to tentatively derive information on the release mechanisms, data were fitted by using the Weibull function^18^

7 $$M_{t}/M_{\inf}=1-e^{-{\left(t/\tau \right)}^{\beta }}$$

where *t* is the time, τ defines the time scale of the process, and β is a shape factor of the release curve. A few best fit curves are shown in Figure 8 while the corresponding τ and β parameters are reported in Table 5. From fitted τ and β, the release rate *dM*_*t*_/*dt* has been obtained: results as a function of time are reported in SI (Figure S9).

**Table 5:** *τ* and *β* Fitted Parameters^a^

conditions	τ (h)	β	matrix structure	release behavior
1× PBS				
1:1, RhB	12.1	1.01	moderately ordered	nearly first-order; smooth, exponential-like profile.
1:2, RhB	9.8	0.98	moderately ordered	controlled and consistent release; close to ideal diffusion.
1:4, RhB	10.9	0.99	moderately ordered	sustained and controlled; matrix structure stable.
1:1, ThT	13.1	0.78	partially ordered	moderately controlled, with a slight initial burst.
1:2, ThT	12.0	0.89	moderately ordered	smooth and sustained, close to exponential.
1:4, ThT	11.7	0.5	highly disordered	fast initial release with reduced control, likely matrix relaxation.
1:1, FITC10	9.6	0.95	moderately ordered	controlled, close to first-order kinetics.
1:4, FITC10	10.3	0.98	moderately ordered	smooth and sustained; nearly exponential profile.
1:1, FITC70	23.2	1.01	well-ordered	highly controlled, very gradual with strong matrix control.
1:4, FITC70	18.5	0.94	moderately ordered	sustained, moderate control with minimal burst.
10× PBS				
1:1, ThT	3.5	0.69	partially ordered	controlled, no sharp burst.
1:2, ThT	1.4	0.45	highly disordered	matrix offers minimal resistance.
1:4, ThT	0.1	0.39	highly disordered	uncontrolled, mimics free diffusion.
1:1, RhB	1.4	0.94	moderately ordered	controlled, diffusion is slowed but smooth.
1:2, RhB	3.8	0.71	partially ordered	quick initially, then slower.
1:4, RhB	0.1	0.30	highly disordered	loose gel, minimal barrier to diffusion.
1:1, FITC10	1.1	0.94	moderately ordered	sustained and consistent diffusion.
1:2, FITC10	1.1	1.10	moderately ordered	smooth and consistent, with slightly accelerating release.
1:4, FITC10	1.1	0.81	partially ordered	moderately fast diffusion with some structural control.
1:1, FITC70	13.8	0.87	moderately ordered	controlled and sustained.
1:2, FITC70	10.0	0.81	partially ordered	sustained release with moderate matrix regulation.
1:4, FITC70	9.9	0.77	partially ordered	gradual and consistent diffusion over time.

a A short comment on the degree of the hydrogel order and the release process is reported. Errors on τ and β are around 30 and 20%, respectively.

Although the Weibull function has been empirically used for the analysis of release kinetics, it has been demonstrated by statistical analysis that the values of β provide a link with the diffusional mechanisms of release.^17,18,43^ In particular, for β values lower than 0.75, the release follows Fickian diffusion; e.g., the release rate is governed by diffusion only and the matrix maintains stable. For Fickian diffusion, the increase of β is associated with a decrease of the disorder of the medium. When β is between 0.75 and 1, anomalous transport due to combination of diffusion and gel swelling occurs while, when β is larger than 1, the diffusion mechanism becomes more complex, and has been described as a combination of phenomena such as diffusion and gel erosion.^17,18^ On the other side, τ is related to the time scale of the release process: in a very simplified way, small τ means fast release while large τ slow release.

In the present case, the release profiles across all probes show marked differences between 10× PBS and 10× PBS conditions. In 10× PBS, release processes show parameters that indicate moderately to well-ordered hydrogel structures (β ≈ 0.9–1.0) and high τ values, which support a sustained and controlled release consistent with Fickian or near-first-order kinetics. These results appear to reflect the degree of structural order of the hydrogel that exerts control over diffusion. τ values were plotted as a function of G-hydrogel diffusion coefficients derived by FRAP experiments (see Figure 9): the clear inversely proportional relationship confirms that the release rate is governed by diffusion only and that the hydrogel matrix stays stable under this condition.

**Figure 9:**
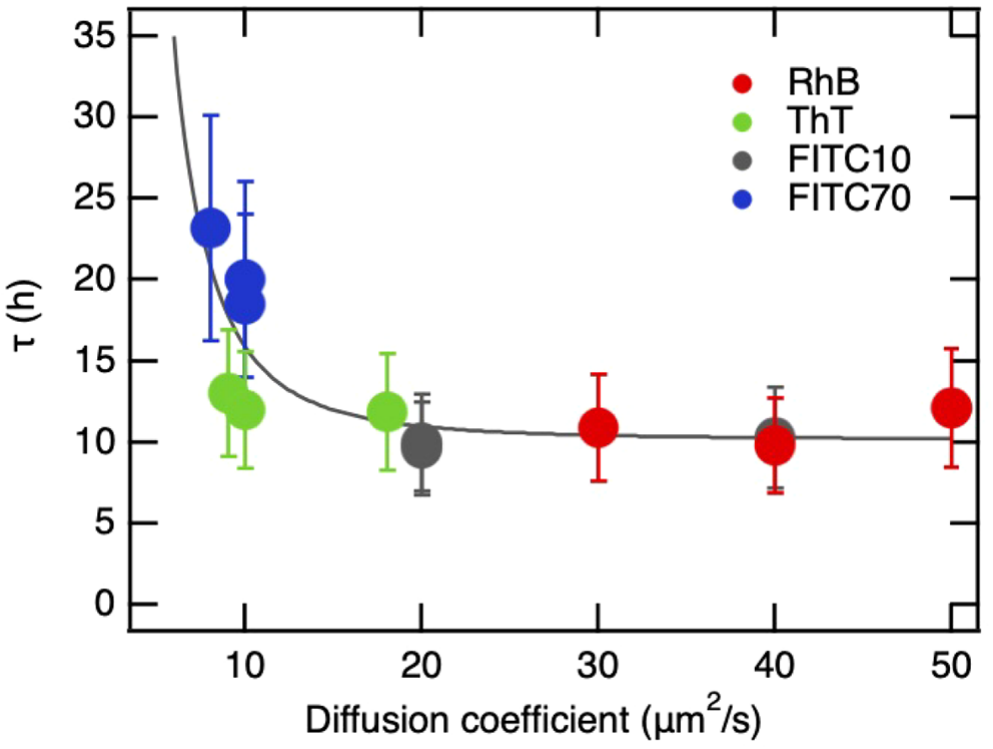
Global dependence of the probe release time constant τ, determined against 1× PBS solution, on diffusion coefficients *D*_c_ observed in G-hydrogels prepared at 95% of water.

In contrast, the release in 10× PBS leads to Weibull pairs that suggest a general disruption of the hydrogel structure, particularly evident in the 1:4 hydrogel, and a transition from slow, sustained Fickian release to rapid anomalous diffusion. Indeed, many systems exhibit very low τ values (e.g., τ = 0.1 for RhB and ThT, but also τ ≈ 1 for FITC10) and β < 0.5, characteristic of highly disordered matrices. Such a general condition reflects in a rapid release, in a few cases mimicking free diffusion, dominated by gel swelling, erosion, or relaxation induced by the different pH or IS of the external 10× PBS solution. The sensitivity of the 1:4 G-hydrogel to such environmental conditions probably leads to a looses structure, reducing its ability to regulate diffusion effectively. It can be concluded that the change from 1:1 to 1:4 composition of the G-hydrogel results in a decrease in structural control in 10× PBS.

The comparison between FITC10 and FITC70 parameters obtained for release in both PBS solutions highlights the influence of molecular weight on diffusion behavior through the G-hydrogel matrix. In both cases, FITC10 displays low τ values, indicating a fast release in agreement with its smaller size and higher mobility. In contrast, FITC70 shows much higher τ values, reflecting a significantly slower release, likely caused by steric hindrance and stronger interactions with the gel network. Noticeable, this effect is still visible in 10× PBS, where mesh loosening and less structural control have been suggested. Despite these differences, β values for both probes in the two solutions remain within a moderately ordered range (0.94–1.1 in 1× PBS and 0.77–1.1 in 10× PBS), suggesting that the matrix structure remains relatively consistent, but interacts differently depending on probe size. Overall, the data confirm that larger probes experience delayed and more restricted diffusion, even in similarly structured or destabilized (by swelling, erosion, or significant relaxation) matrices.

## Conclusions

In supramolecular physical hydrogels, the nature of the weak interactions responsible for the formation of the entangled network controls the transport processes in, through, and from the gel.^44,45^

This study provides a comprehensive analysis of the transport properties of G-hydrogels formed by the self-assembly of guanosine and guanosine monophosphate. By using a combination of FRAP, time-resolved fluorescence spectroscopy, UV–vis titration, and SAXS/WAXS techniques, we demonstrated that the diffusion behavior of various solutes within the G-hydrogel matrix is highly tunable and predominantly governed by a Fickian mechanism under the appropriate conditions. The hydrogel structure remains stable across different compositions and probe loadings, as confirmed by scattering data, with the mesh size and network entanglement (e.g., stiffness and stickiness) directly controlled by the Gua:GMP molar ratio and water content. Hydrophilic probes, such as FITC-dextrans and RhB, exhibited diffusion behavior consistent with obstruction and free volume theories, whereas amphiphilic probes such as ThT and DAPI showed reduced mobility due to significant binding interactions with the G-quadruplex framework. The multiscale diffusion model (MSDM) successfully described the observed diffusion coefficients, correlating the molecular probe size with network properties. Release experiments further confirmed that in physiological-like conditions (1× PBS), the solute release kinetics align with a sustained, Fickian-controlled regime, while higher ionic strength (10× PBS) destabilizes the network, leading to faster and less controlled diffusion. In conclusion, the results highlight the excellent tunability of G-hydrogels, making them promising candidates for applications in drug delivery, controlled release, and biomedical scaffolding, where the precise control of molecular transport is critical.

## Supplementary Material



## Data Availability

Data for this article will be made public and available at Zenodo repository.

## Notes

The content is: molecular structures of the dyes (Figure S1); UV–vis absorption spectroscopy results for ThT in hydrogel 1:4 95% prepared at different IS (Figure S2); SAXS/WAXS profiles for hydrogel 1:4 95% prepared at different ThT concentration (Figure S3); UV–vis absorption spectroscopy whole results (Figures S4 and S5); diffusion coefficients obtained by FRAP experiments plotted as a function of the MW of the probes (Figure S6); schematic models for the network (Figure S7); flourescence images observed during a probe loading experiment as a function of time (Figure S8); the probe release rate obtained by Weibull approach (Figure S9) (PDF)
