# Supplementary material for: Transport Properties of Self-Assembling G‑Hydrogels: Evidence for a Tunable Fickian Diffusivity
Source: J Phys Chem B. 2025 May 15;129(21):5136–49. doi: 10.1021/acs.jpcb.5c00564 (PMC12128034; doi:10.1021/acs.jpcb.5c00564)
Supplement: Supplementary file 1 [file jp5c00564_si_001.pdf]

Supporting Information: Transport properties of self-assembling G-hydrogels: evidences for a tunable Fickian diffusivity

Alessia Pepe,[*,†] Paolo Moretti,[†] Paolo Mariani,[*,†] Valentina Notarstefano,[‡] and Francesca Ripanti[†]

†*Università Politecnica delle Marche, Department of Life and Environmental Sciences, 60131 Ancona, Italy*

‡*Università di Teramo, Department of Science and Technology for Agriculture, Food and Environment, 64100 Teramo, Italy*

E-mail: a.pepe@univpm.it; p.mariani@univpm.it

Fig. S1: Molecular structure of the fluorescent dyes

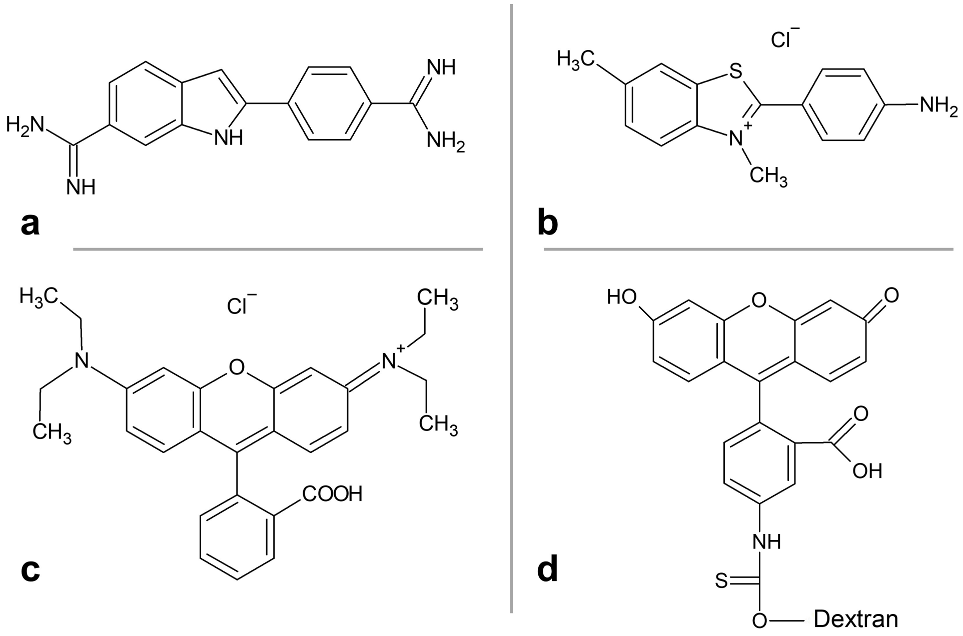

Figure S1: Molecular structure of: (a) DAPI, (b) ThT, (c) RhB, (d) FITC-dextran.

Fig. S2: UV-Vis absorption for ThT in 1:4 95% G-hydrogel at different Ionic Strength (IS)

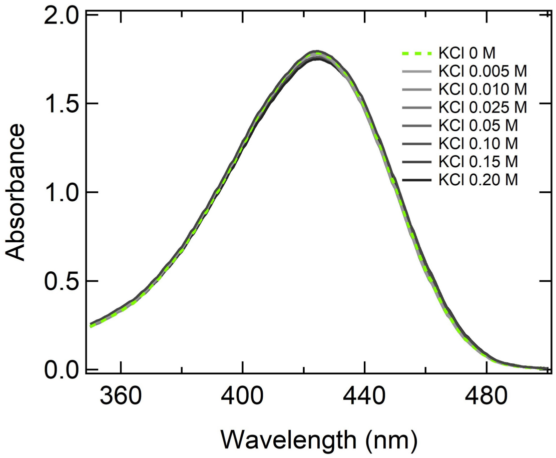

Figure S2: Absorption spectra of samples containing constant values of G-hydrogel 1:4 95% and ThT and different concentrations of KCl. The dot curve is related to the sample prepared without the addition of KCl, while the other ones regard KCl concentration ranging from 0.005 to 0.2 M.

Fig. S3: SAXS and WAXS results for 1:4 98% G-hydrogel at different ThT concentration

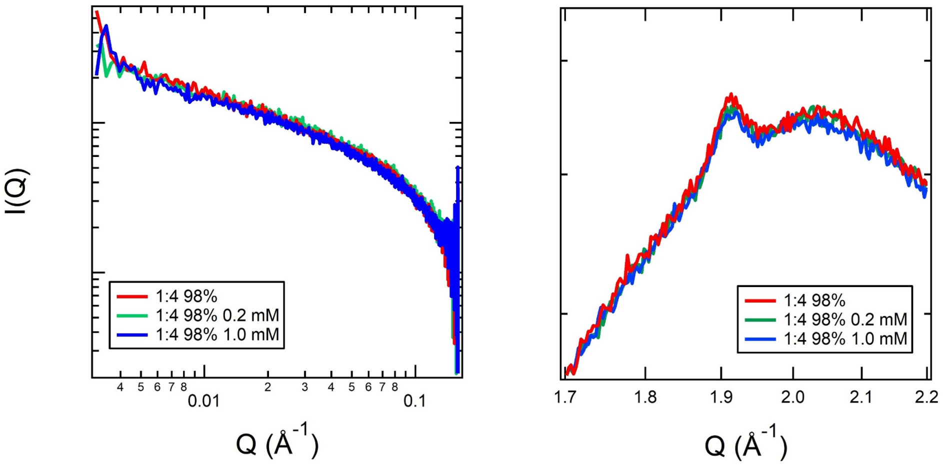

Figure S3: SAXS (left panel) and WAXS (right panel) profiles of G-hydrogels prepared considering two higher concentrations of ThT than previously described. Red curves refer to empty G-hydrogel 1:4 98%, green curves to G-hydrogel with 0.2 mM of ThT and blue ones to G-hydrogel with 1.0 mM of ThT.

Fig.s S4 and S5: UV-Vis absorption results

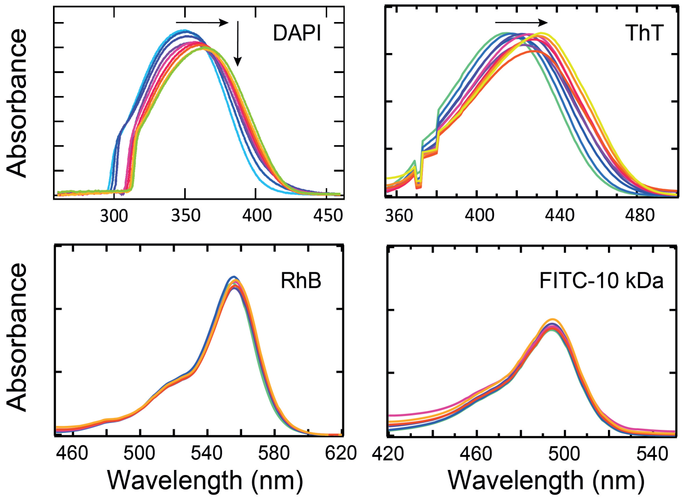

Figure S4: Absorption spectra measured during G-hydrogel titration of the solutions containing DAPI, ThT, RhB, and FITC-dextran 10 kDa.

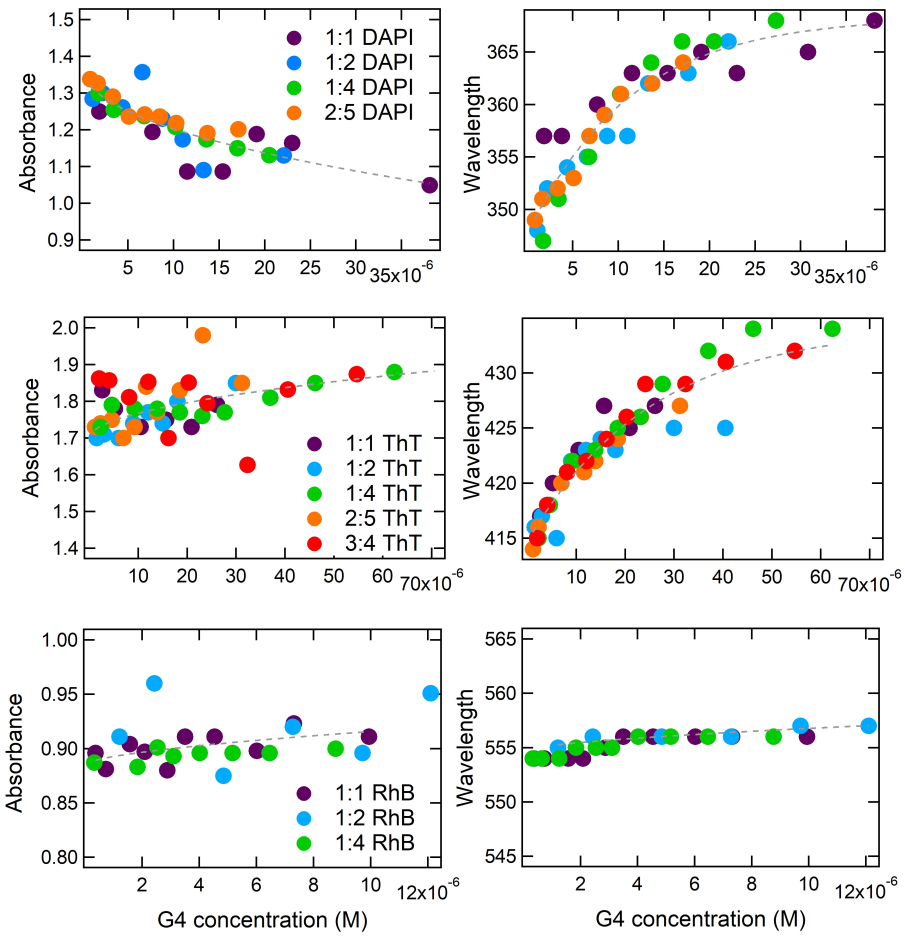

Figure S5: Changes in the absorption spectra (absorption intensity on the left and wavelength of the absorption maximum on the right) during titration of G-hydrogels to the different probe solutions (DAPI, first row; ThT, second row; RhB, third row).

Fig. S6: FRAP results

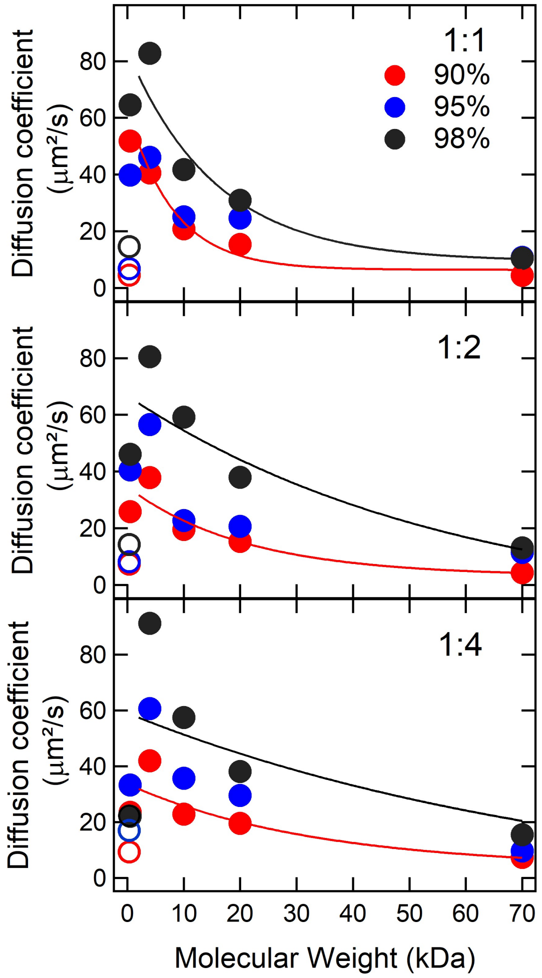

Figure S6: Dependence of diffusion coefficients on molecular weight for all the used probes. Since the ThT coefficients differ from those derived for the other probes (rather small compared to the ThT MW), their position in the graph is highlighted using empty dots as symbols. Lines, shown only for 1:1 and 1:4 cases, are guide for the eye.

Fig. S7: Schematic mesh structure

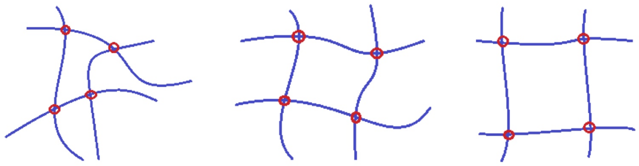

Figure S7: Simple model representation of the network mesh in G-hydrogels. From the left: low-charged, flexible quadruplexes, as in 1:1 G-hydrogels; charged and rather rigid quadruplexes, as in 1:2 G-hydrogels; very charged and rigid quadruplexes, as in 1:4 G-hydrogels.

Fig. S8: Solute loading in G-hydrogels inside a glass capillary

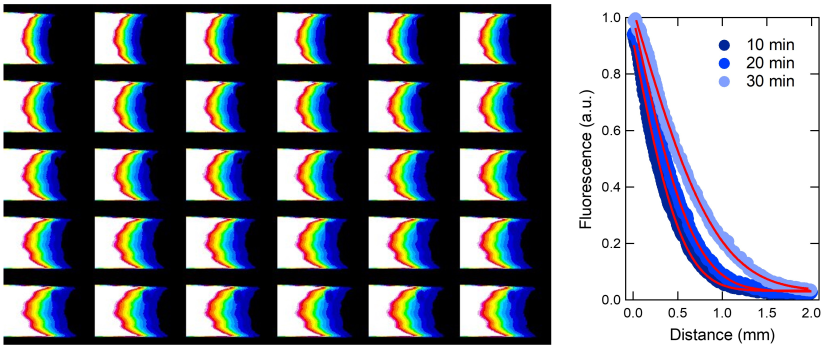

Figure S8: On the left: propagation of the FITC-dextran 4 kDa fluorescence inside a 1:4 98% G-hydrogel, as measured in a capillary at the gel/solution interface position. Frames were collected every minute for 30 min. In the used color map, white indicates the fluorescence of the probe in the reservoir solution, while colors from red to black correlate with the decay of fluorescence intensity when entering the G-hydrogel. Capillary diameter was 1 mm. On the right: dependence of the FITC-dextran normalized fluorescence intensity on the distance from the solution/G-hydrogel interface. The three curves refer to different time frames (see left panel), as indicated. Lines are best fit to the data by Eq. 6 of the main text.

Fig. S9: Probe release rate

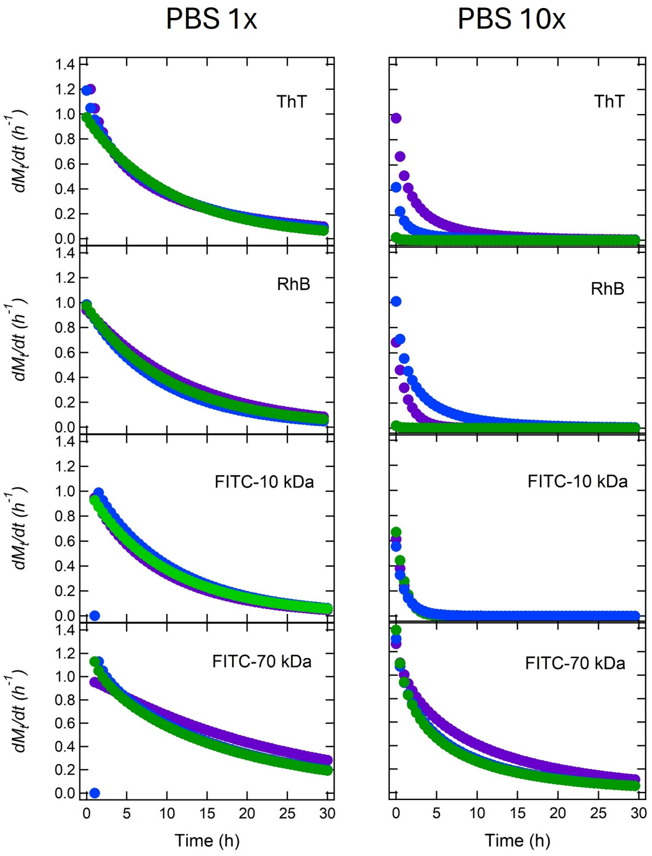

Figure S9: Release rate calculated from the Weibull parameters τ and β obtained for ThT, RhB, FITC10 and FITC70 released form G-hydrogel prepared at 95% and 1:1 (purple curves), 1:2 (cyan curves), and 1:4 (green curves) Gua:GMP molar ratios. Experiments were performed against 1x and 10x PBS solutions.
